# [μ-Bis(diphenyl­arsino)methane-1:2κ^2^ *As*:*As*′]nona­carbonyl-1κ^3^ *C*,2κ^3^ *C*,3κ^3^ *C*-tricyclo­hexyl­phosphine-3κ*P*-*triangulo*-triruthenium(0)

**DOI:** 10.1107/S1600536809047977

**Published:** 2009-11-21

**Authors:** Omar bin Shawkataly, Imthyaz Ahmed Khan, Chin Sing Yeap, Hoong-Kun Fun

**Affiliations:** a Chemical Sciences Programme, School of Distance Education, Universiti Sains Malaysia, 11800 USM, Penang, Malaysia; b X-ray Crystallography Unit, School of Physics, Universiti Sains Malaysia, 11800 USM, Penang, Malaysia

## Abstract

In the title *triangulo*-triruthenium compound, [Ru_3_(C_25_H_22_As_2_)(C_18_H_33_P)(CO)_9_], the bis­(diphenyl­arsino)methane ligand bridges an Ru—Ru bond and the monodentate phosphine ligand bonds to the third Ru atom. Both the phosphine and arsine ligands are equatorial with respect to the Ru_3_ triangle. In addition, each Ru atom carries one equatorial and two axial terminal carbonyl ligands. All three cyclo­hexane rings are disordered over two positions with site occupancies of 0.628 (6) and 0.372 (6). The mean planes of these three phosphine-substituted cyclo­hexane rings make dihedral angles of 53.0 (8), 68.3 (6) and 89.9 (7)° (major components), and 46.7 (14), 41.3 (11) and 75.8 (10)° (minor components) with each other. The dihedral angles between the two phenyl rings are 85.0 (2) and 88.1 (2)° for the two diphenyl­arsino groups. Two cyclo­hexane rings adopt a chair conformation whereas the other adopts a slightly twisted chair conformation for the major components; these conformations are similiar for the minor components. Intra­molecular C—H⋯O hydrogen bonds stabilize the mol­ecular structure. In the crystal packing, the mol­ecules are linked together into chains *via* inter­molecular C—H⋯O hydrogen bonds down the *a* axis. Weak inter­molecular C—H⋯π inter­actions further stabilize the crystal structure.

## Related literature

For general background to *triangulo*-triruthenium derivatives, see: Bruce *et al.* (1985 ▶, 1988*a* ▶,*b* ▶); Shawkataly *et al.* (1998 ▶, 2004 ▶). For related structures, see: Shawkataly *et al.* (2009*a* ▶,*b* ▶). For the synthesis of bis­(diphenylarsino)methane, see: Bruce *et al.* (1983 ▶). For ring conformations, see: Cremer & Pople (1975 ▶). For the stability of the temperature controller used for the data collection, see: Cosier & Glazer (1986 ▶).
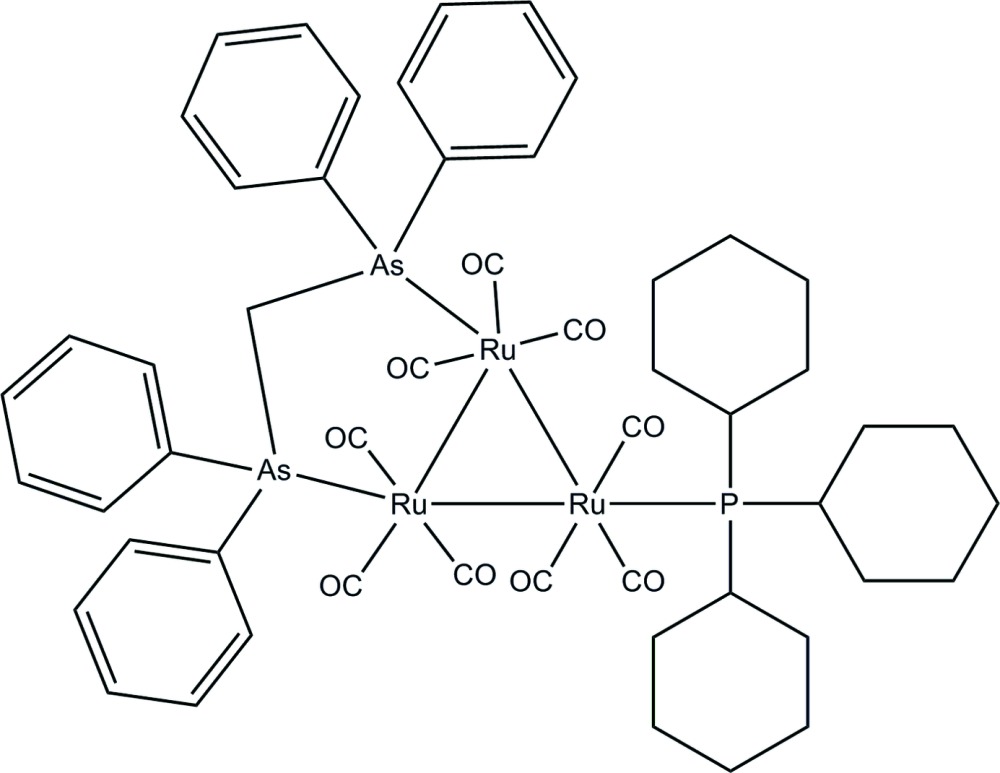


## Experimental

### 

#### Crystal data

[Ru_3_(C_25_H_22_As_2_)(C_18_H_33_P)(CO)_9_]
*M* *_r_* = 1307.98
Orthorhombic, 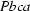

*a* = 15.6489 (2) Å
*b* = 20.9223 (3) Å
*c* = 31.3447 (5) Å
*V* = 10262.6 (3) Å^3^
*Z* = 8
Mo *K*α radiation
μ = 2.24 mm^−1^
*T* = 100 K
0.30 × 0.14 × 0.04 mm

#### Data collection

Bruker SMART APEXII CCD area-detector diffractometer
Absorption correction: multi-scan (**SADABS**; Bruker, 2005 ▶) *T*_min_ = 0.555, *T*_max_ = 0.922
62224 measured reflections
11768 independent reflections
8148 reflections with *I* > 2σ(*I*)
*R*_int_ = 0.092

#### Refinement

*R*[*F* ^2^ > 2σ(*F* ^2^)] = 0.046
*wR*(*F* ^2^) = 0.099
*S* = 1.01
11768 reflections
728 parameters
379 restraints
H-atom parameters constrained
Δρ_max_ = 0.84 e Å^−3^
Δρ_min_ = −0.61 e Å^−3^

### 

Data collection: *APEX2* (Bruker, 2005 ▶); cell refinement: *SAINT* (Bruker, 2005 ▶); data reduction: *SAINT*; program(s) used to solve structure: *SHELXTL* (Sheldrick, 2008 ▶); program(s) used to refine structure: *SHELXTL*; molecular graphics: *SHELXTL*; software used to prepare material for publication: *SHELXTL* and *PLATON* (Spek, 2009 ▶).

## Supplementary Material

Crystal structure: contains datablocks global, I. DOI: 10.1107/S1600536809047977/sj2677sup1.cif

Structure factors: contains datablocks I. DOI: 10.1107/S1600536809047977/sj2677Isup2.hkl

Additional supplementary materials:  crystallographic information; 3D view; checkCIF report

## Figures and Tables

**Table 1:** Hydrogen-bond geometry (Å, °)

*D*—H⋯*A*	*D*—H	H⋯*A*	*D*⋯*A*	*D*—H⋯*A*
C31*A*—H31*B*⋯O8	0.97	2.56	3.481 (17)	159
C32*A*—H32*A*⋯O9	0.98	2.47	3.157 (14)	127
C43*A*—H43*B*⋯O9^i^	0.97	2.18	2.954 (10)	135
C5—H5*A*⋯*Cg*1^ii^	0.93	2.94	3.728 (5)	144
C10—H10*A*⋯*Cg*2^iii^	0.93	2.90	3.651 (5)	139
C16—H16*A*⋯*Cg*2^iv^	0.93	2.96	3.718 (5)	140
C22—H22*A*⋯*Cg*1^v^	0.93	2.82	3.608 (5)	143
C41*B*—H41*D*⋯*Cg*3^ii^	0.97	2.62	3.420 (14)	140

Symmetry codes: (i) 
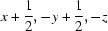
; (ii) 
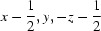
; (iii) 
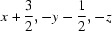
; (iv) 
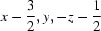
; (v) 
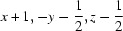
. *Cg*1, *Cg*2 and *Cg*3 are the centroids of the C7–C12, C20–C25 and C1–C6 phenyl rings, respectively.

**Fig. 1.**
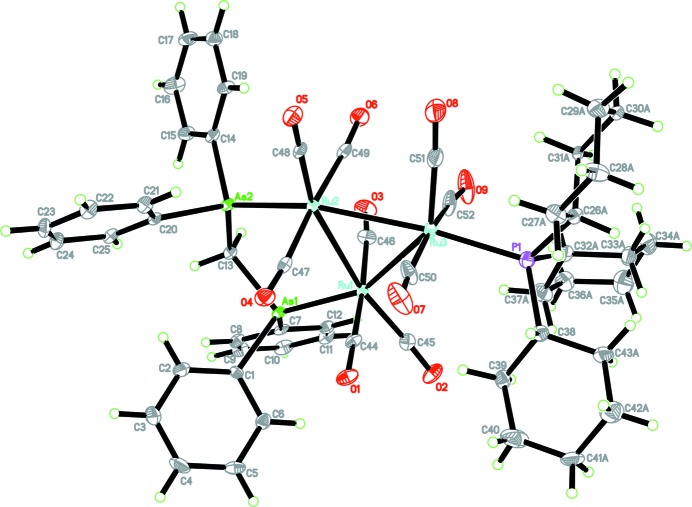
The molecular structure of the title compound with 30% probability ellipsoids for non-H atoms. Only the major components are shown.

**Fig. 2.**
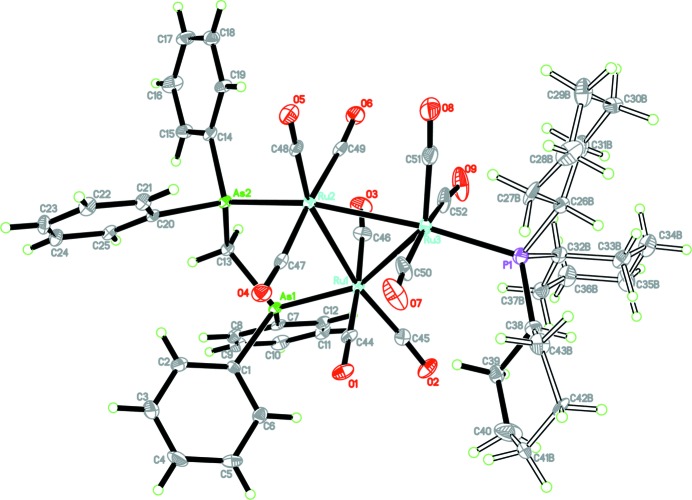
The molecular structure of the title compound with 30% probability ellipsoids for non-H atoms. Only the minor components are shown.

**Fig. 3.**
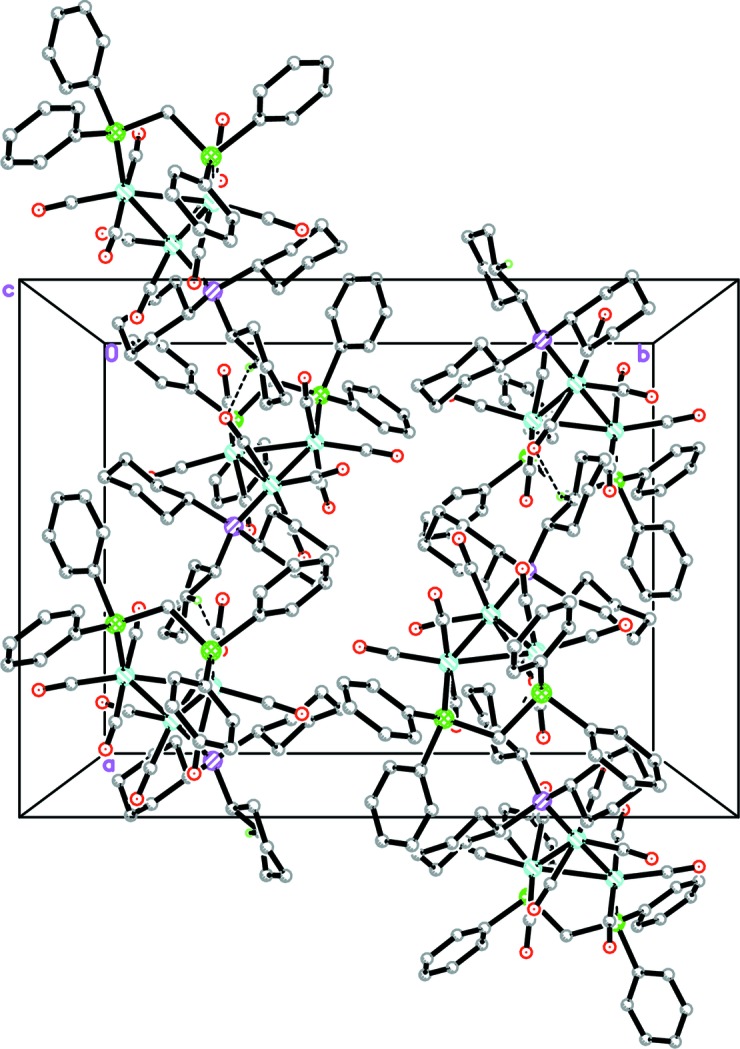
The crystal packing of the title compound, viewed down the c axis, showing the molecules linked down a axis. Hydrogen atoms that not involved in the hydrogen-bonding (dashed lines) have been omitted for clarity. Only the major components are shown.

## supplementary crystallographic information

### Comment

Tri-angulotriruthenium clusters are known for their interesting structural variations and related catalytic activity. A large number of substituted derivatives, Ru_3_(CO)_12-n_*L*_n_ (*L* = group 15 ligand) have been reported (Bruce *et al.*, 1985, 1988a,b). As part of our study on the substitution of transition metal-carbonyl clusters with mixed-ligand complexes, we have published several structures of tri-angulotriruthenium-carbonyl clusters containing mixed P/As and P/Sb ligands (Shawkataly *et al.*, 1998, 2004, 2009a,b). Herein we report the synthesis and structure of Ru_3_(C_18_H_33_P)(C_25_H_22_As_2_)(CO)_9_.

The bond lengths and angles of title compound (Fig. 1 & 2) are comparable to those in related structures (Shawkataly *et al.*, 2009a,b). The bis(diphenylarsino)methane ligand bridges the Ru1—Ru2 bond and the monodentate phosphine ligand bonds to the Ru3 atom. Both the phosphine and arsine ligands are equatorial with respect to the Ru_3_ triangle. Additionally, each Ru atom carries one equatorial and two axial terminal carbonyl ligands. All three cyclohexane rings are disordered over two positions with site occupancies of 0.628 (6) and 0.372 (6). Only the C41, C42 and C43 atoms in the C38-C43 ring are disordered. The mean planes of these three phosphine-substituted cyclohexane rings make dihedral angles (C26–C31/C32–C37, C26–C31/C38–C43 and C32–C37/C38–C43) of 53.0 (8), 68.3 (6) and 89.9 (7)° with each other respectively for the major components and 46.7 (14), 41.3 (11) and 75.8 (10)° for the minor components. The dihedral angles between the two phenyl rings (C1–C6/C7–C12 and C14–C19/C20–C25) are 85.0 (2) and 88.1 (2)° for the two diphenylarsino groups respectively. Four cyclohexane rings adopt a chair conformation with puckering amplitude Q = 0.555 (17) Å, θ = 0.0 (18)°, φ = 191 (176)° for C26A–C32A ring, Q = 0.572 (18) Å, θ = 180.0 (18)°, φ = 235 (45)° for C32A–C37A ring, Q = 0.52 (3) Å, θ = 10 (3)°, φ = 120 (26)° for C26B–C31B ring and Q = 0.58 (3) Å, θ = 177 (3)°, φ = 90 (166)° for C32B–C37B ring. The other two cyclohexane rings (C38–C43A and C38–C43B) adopt a slightly twisted chair conformation with Q = 0.545 (8) Å, θ = 159.3 (8)°, φ = 137 (2)° and Q = 0.465 (12) Å, θ = 27.0 (14)°, φ = 307 (3)°, respectively (Cremer & Pople, 1975).

In the crystal packing (Fig. 3), intramolecular C31—H31B···O8 and C32—H32A···O9 hydrogen bonds stabilize the molecular structure and the molecules are linked together into chains *via* intermolecular C43—H43B···O9 hydrogen bonds down *a* axis. Weak intermolecular C—H···π interactions further stabilize the crystal structure (Table 1).

### Experimental

The reactions were conducted under an atmosphere of nitrogen using standard Schlenk techniques and hexane-dried over sodium metal. Tricyclohexylphosphine (Strem Chemicals) used as received and bis(diphenylarsino)methane (Bruce *et al.*, 1983) was prepared by the reported procedure. The title compound was obtained by refluxing equimolar quantities of Ru_3_(CO)_10_(µ-Ph_2_AsCH_2_AsPh_2_) (105.5 mg, 0.1 mmol) and tricyclohexylphosphine (27.8 mg, 0.1 mmol) in hexane under nitrogen atmosphere. Crystals suitable for X-ray diffraction were grown by slow solvent / solvent diffusion of CH_3_OH into CHCl_3_.

### Refinement

All three cyclohexane rings are disordered over two positions. The refined site occupancies are about the same and in the final refinement these site occupancies are made to be the same and refined to 0.628 (6) and 0.372 (6). The same U^ij^ parameters is used for the atom pair C26A/C26B and C32A/C32B. The C26A–C37B atoms were subjected to rigid bond and similarity restraints. All hydrogen atoms were positioned geometrically and refined using a riding model with C—H = 0.93–0.98 Å and *U*_iso_(H) = 1.2 *U*_eq_(C).

### Crystal data

**Table:**

[Ru_3_(C_25_H_22_As_2_)(C_18_H_33_P)(CO)_9_]	*F*(000) = 5216
*M**_r_* = 1307.98	*D*_x_ = 1.693 Mg m^−^^3^
Orthorhombic, *P**b**c**a*	Mo *K*α radiation, λ = 0.71073 Å
Hall symbol: -P 2ac 2ab	Cell parameters from 9061 reflections
*a* = 15.6489 (2) Å	θ = 2.3–26.4°
*b* = 20.9223 (3) Å	µ = 2.24 mm^−^^1^
*c* = 31.3447 (5) Å	*T* = 100 K
*V* = 10262.6 (3) Å^3^	Plate, red
*Z* = 8	0.30 × 0.14 × 0.04 mm

### Data collection

**Table:**

Bruker SMART APEXII CCD area-detector diffractometer	11768 independent reflections
Radiation source: fine-focus sealed tube	8148 reflections with *I* > 2σ(*I*)
graphite	*R*_int_ = 0.092
φ and ω scans	θ_max_ = 27.5°, θ_min_ = 2.1°
Absorption correction: multi-scan (*SADABS*; Bruker, 2005)	*h* = −17→20
*T*_min_ = 0.555, *T*_max_ = 0.922	*k* = −27→27
62224 measured reflections	*l* = −34→40

### Refinement

**Table:**

Refinement on *F*^2^	Primary atom site location: structure-invariant direct methods
Least-squares matrix: full	Secondary atom site location: difference Fourier map
*R*[*F*^2^ > 2σ(*F*^2^)] = 0.046	Hydrogen site location: inferred from neighbouring sites
*wR*(*F*^2^) = 0.099	H-atom parameters constrained
*S* = 1.01	*w* = 1/[σ^2^(*F*_o_^2^) + (0.0381*P*)^2^] where *P* = (*F*_o_^2^ + 2*F*_c_^2^)/3
11768 reflections	(Δ/σ)_max_ = 0.001
728 parameters	Δρ_max_ = 0.84 e Å^−^^3^
379 restraints	Δρ_min_ = −0.61 e Å^−^^3^

### Special details

**Table:**

Experimental. The crystal was placed in the cold stream of an Oxford Cyrosystems Cobra open-flow nitrogen cryostat (Cosier & Glazer, 1986) operating at 100.0 (1) K.
Geometry. All e.s.d.'s (except the e.s.d. in the dihedral angle between two l.s. planes) are estimated using the full covariance matrix. The cell e.s.d.'s are taken into account individually in the estimation of e.s.d.'s in distances, angles and torsion angles; correlations between e.s.d.'s in cell parameters are only used when they are defined by crystal symmetry. An approximate (isotropic) treatment of cell e.s.d.'s is used for estimating e.s.d.'s involving l.s. planes.
Refinement. Refinement of *F*^2^ against ALL reflections. The weighted *R*-factor *wR* and goodness of fit *S* are based on *F*^2^, conventional *R*-factors *R* are based on *F*, with *F* set to zero for negative *F*^2^. The threshold expression of *F*^2^ > σ(*F*^2^) is used only for calculating *R*-factors(gt) *etc*. and is not relevant to the choice of reflections for refinement. *R*-factors based on *F*^2^ are statistically about twice as large as those based on *F*, and *R*- factors based on ALL data will be even larger.

### Fractional atomic coordinates and isotropic or equivalent isotropic displacement parameters (Å^2^)

**Table:**

	*x*	*y*	*z*	*U*_iso_*/*U*_eq_	Occ. (<1)
Ru1	0.78575 (2)	0.257325 (16)	0.150385 (12)	0.01745 (9)	
Ru2	0.76356 (2)	0.392912 (16)	0.142057 (12)	0.01658 (9)	
Ru3	0.86145 (2)	0.324628 (17)	0.079835 (12)	0.02083 (10)	
As1	0.70760 (3)	0.25391 (2)	0.216742 (15)	0.01608 (11)	
As2	0.65368 (3)	0.40103 (2)	0.196880 (15)	0.01603 (11)	
P1	0.95236 (9)	0.26464 (7)	0.03344 (4)	0.0311 (3)	
O1	0.9585 (2)	0.28141 (15)	0.19389 (12)	0.0305 (8)	
O2	0.8418 (2)	0.11900 (15)	0.14390 (12)	0.0379 (9)	
O3	0.6058 (2)	0.24373 (17)	0.11196 (12)	0.0403 (10)	
O4	0.9075 (2)	0.41492 (15)	0.20650 (12)	0.0336 (9)	
O5	0.7950 (2)	0.52994 (15)	0.11409 (11)	0.0335 (9)	
O6	0.6264 (2)	0.37817 (17)	0.07316 (12)	0.0396 (10)	
O7	1.0200 (3)	0.3757 (2)	0.12621 (13)	0.0594 (13)	
O8	0.8284 (3)	0.44135 (18)	0.02666 (14)	0.0682 (15)	
O9	0.7173 (3)	0.2515 (2)	0.03533 (14)	0.0651 (14)	
C1	0.7736 (3)	0.25922 (19)	0.26884 (14)	0.0165 (10)	
C2	0.7567 (4)	0.3011 (2)	0.30151 (18)	0.0402 (14)	
H2A	0.7106	0.3290	0.2994	0.048*	
C3	0.8079 (4)	0.3022 (3)	0.33755 (19)	0.0574 (19)	
H3A	0.7958	0.3309	0.3594	0.069*	
C4	0.8763 (4)	0.2614 (3)	0.34129 (17)	0.0386 (14)	
H4A	0.9109	0.2627	0.3654	0.046*	
C5	0.8932 (3)	0.2191 (2)	0.30948 (17)	0.0300 (12)	
H5A	0.9389	0.1909	0.3121	0.036*	
C6	0.8426 (3)	0.2176 (2)	0.27293 (16)	0.0261 (11)	
H6A	0.8549	0.1887	0.2512	0.031*	
C7	0.6337 (3)	0.17998 (19)	0.22765 (15)	0.0199 (10)	
C8	0.5879 (3)	0.1771 (2)	0.26535 (16)	0.0229 (11)	
H8A	0.5938	0.2090	0.2858	0.027*	
C9	0.5333 (3)	0.1262 (2)	0.27232 (17)	0.0276 (12)	
H9A	0.5025	0.1241	0.2976	0.033*	
C10	0.5239 (3)	0.0788 (2)	0.24239 (18)	0.0304 (13)	
H10A	0.4871	0.0448	0.2475	0.036*	
C11	0.5692 (3)	0.0816 (2)	0.20470 (17)	0.0282 (12)	
H11A	0.5628	0.0495	0.1844	0.034*	
C12	0.6248 (3)	0.1325 (2)	0.19694 (16)	0.0216 (10)	
H12A	0.6555	0.1346	0.1716	0.026*	
C13	0.6179 (3)	0.31912 (18)	0.22109 (15)	0.0180 (10)	
H13A	0.5673	0.3043	0.2062	0.022*	
H13B	0.6031	0.3251	0.2509	0.022*	
C14	0.5457 (3)	0.4367 (2)	0.17684 (15)	0.0204 (10)	
C15	0.4676 (3)	0.4199 (2)	0.19500 (16)	0.0272 (12)	
H15A	0.4653	0.3888	0.2162	0.033*	
C16	0.3936 (3)	0.4497 (2)	0.18139 (18)	0.0372 (14)	
H16A	0.3415	0.4391	0.1938	0.045*	
C17	0.3970 (3)	0.4950 (2)	0.14949 (17)	0.0333 (13)	
H17A	0.3470	0.5146	0.1402	0.040*	
C18	0.4738 (3)	0.5115 (2)	0.13144 (16)	0.0281 (12)	
H18A	0.4758	0.5418	0.1098	0.034*	
C19	0.5482 (3)	0.4830 (2)	0.14539 (15)	0.0237 (11)	
H19A	0.6003	0.4949	0.1336	0.028*	
C20	0.6707 (3)	0.45555 (19)	0.24663 (15)	0.0181 (10)	
C21	0.7242 (3)	0.5090 (2)	0.24203 (16)	0.0235 (11)	
H21A	0.7543	0.5154	0.2168	0.028*	
C22	0.7318 (3)	0.5521 (2)	0.27545 (16)	0.0268 (12)	
H22A	0.7671	0.5876	0.2725	0.032*	
C23	0.6874 (3)	0.5430 (2)	0.31291 (16)	0.0260 (11)	
H23A	0.6928	0.5723	0.3350	0.031*	
C24	0.6351 (3)	0.4907 (2)	0.31771 (16)	0.0274 (11)	
H24A	0.6059	0.4844	0.3432	0.033*	
C25	0.6257 (3)	0.4470 (2)	0.28446 (15)	0.0212 (10)	
H25A	0.5894	0.4121	0.2876	0.025*	
C26A	0.9933 (11)	0.3072 (5)	−0.0121 (6)	0.024 (2)	0.628 (6)
H26A	1.0298	0.2778	−0.0283	0.029*	0.628 (6)
C27A	1.0489 (7)	0.3655 (5)	0.0017 (4)	0.031 (3)	0.628 (6)
H27A	1.0156	0.3930	0.0202	0.037*	0.628 (6)
H27B	1.0979	0.3504	0.0177	0.037*	0.628 (6)
C28A	1.0789 (8)	0.4027 (5)	−0.0358 (4)	0.036 (3)	0.628 (6)
H28A	1.1174	0.3763	−0.0524	0.043*	0.628 (6)
H28B	1.1110	0.4394	−0.0258	0.043*	0.628 (6)
C29A	1.0077 (13)	0.4257 (6)	−0.0644 (6)	0.034 (4)	0.628 (6)
H29A	1.0316	0.4471	−0.0891	0.041*	0.628 (6)
H29B	0.9723	0.4562	−0.0491	0.041*	0.628 (6)
C30A	0.9523 (14)	0.3681 (9)	−0.0790 (5)	0.022 (3)	0.628 (6)
H30A	0.9042	0.3836	−0.0955	0.026*	0.628 (6)
H30B	0.9862	0.3406	−0.0972	0.026*	0.628 (6)
C31A	0.9203 (10)	0.3305 (8)	−0.0423 (5)	0.018 (3)	0.628 (6)
H31A	0.8892	0.2937	−0.0529	0.022*	0.628 (6)
H31B	0.8807	0.3566	−0.0261	0.022*	0.628 (6)
C32A	0.8926 (8)	0.1935 (6)	0.0039 (5)	0.025 (2)	0.628 (6)
H32A	0.8396	0.2123	−0.0071	0.029*	0.628 (6)
C33A	0.9359 (8)	0.1631 (6)	−0.0349 (4)	0.033 (3)	0.628 (6)
H33A	0.9516	0.1962	−0.0552	0.040*	0.628 (6)
H33B	0.9878	0.1415	−0.0260	0.040*	0.628 (6)
C34A	0.8764 (8)	0.1152 (7)	−0.0565 (4)	0.046 (3)	0.628 (6)
H34A	0.9056	0.0961	−0.0806	0.055*	0.628 (6)
H34B	0.8265	0.1375	−0.0672	0.055*	0.628 (6)
C35A	0.8476 (11)	0.0620 (7)	−0.0259 (6)	0.054 (4)	0.628 (6)
H35A	0.8068	0.0343	−0.0400	0.065*	0.628 (6)
H35B	0.8964	0.0364	−0.0173	0.065*	0.628 (6)
C36A	0.8073 (14)	0.0918 (9)	0.0125 (6)	0.041 (3)	0.628 (6)
H36A	0.7926	0.0584	0.0326	0.050*	0.628 (6)
H36B	0.7546	0.1127	0.0039	0.050*	0.628 (6)
C37A	0.8636 (17)	0.1400 (10)	0.0345 (5)	0.040 (4)	0.628 (6)
H37A	0.9135	0.1185	0.0460	0.048*	0.628 (6)
H37B	0.8326	0.1588	0.0582	0.048*	0.628 (6)
C26B	0.996 (2)	0.3247 (10)	−0.0109 (10)	0.024 (2)	0.372 (6)
H26B	1.0453	0.3027	−0.0235	0.029*	0.372 (6)
C27B	1.0324 (13)	0.3903 (8)	0.0049 (7)	0.037 (4)	0.372 (6)
H27C	0.9876	0.4139	0.0194	0.044*	0.372 (6)
H27D	1.0782	0.3828	0.0251	0.044*	0.372 (6)
C28B	1.0648 (13)	0.4284 (7)	−0.0310 (8)	0.042 (5)	0.372 (6)
H28C	1.1122	0.4057	−0.0439	0.051*	0.372 (6)
H28D	1.0870	0.4684	−0.0198	0.051*	0.372 (6)
C29B	1.000 (2)	0.4435 (13)	−0.0653 (10)	0.042 (5)	0.372 (6)
H29C	1.0276	0.4671	−0.0882	0.051*	0.372 (6)
H29D	0.9548	0.4696	−0.0536	0.051*	0.372 (6)
C30B	0.963 (3)	0.3795 (17)	−0.0826 (9)	0.029 (5)	0.372 (6)
H30C	0.9158	0.3889	−0.1015	0.034*	0.372 (6)
H30D	1.0070	0.3581	−0.0993	0.034*	0.372 (6)
C31B	0.9339 (19)	0.3363 (17)	−0.0491 (10)	0.033 (6)	0.372 (6)
H31C	0.9213	0.2953	−0.0621	0.040*	0.372 (6)
H31D	0.8808	0.3530	−0.0378	0.040*	0.372 (6)
C32B	0.9148 (16)	0.2027 (11)	0.0066 (9)	0.025 (2)	0.372 (6)
H32B	0.8631	0.2197	−0.0069	0.029*	0.372 (6)
C33B	0.9658 (12)	0.1758 (11)	−0.0309 (8)	0.031 (4)	0.372 (6)
H33C	1.0165	0.1544	−0.0202	0.037*	0.372 (6)
H33D	0.9842	0.2107	−0.0491	0.037*	0.372 (6)
C34B	0.9128 (13)	0.1285 (10)	−0.0572 (7)	0.036 (4)	0.372 (6)
H34C	0.9479	0.1115	−0.0800	0.043*	0.372 (6)
H34D	0.8652	0.1510	−0.0701	0.043*	0.372 (6)
C35B	0.8781 (18)	0.0727 (10)	−0.0304 (9)	0.042 (5)	0.372 (6)
H35C	0.8413	0.0459	−0.0477	0.050*	0.372 (6)
H35D	0.9249	0.0468	−0.0198	0.050*	0.372 (6)
C36B	0.828 (2)	0.1003 (15)	0.0064 (10)	0.039 (5)	0.372 (6)
H36C	0.8080	0.0658	0.0244	0.047*	0.372 (6)
H36D	0.7787	0.1229	−0.0046	0.047*	0.372 (6)
C37B	0.881 (3)	0.1458 (16)	0.0329 (8)	0.033 (5)	0.372 (6)
H37C	0.9293	0.1228	0.0449	0.040*	0.372 (6)
H37D	0.8468	0.1617	0.0563	0.040*	0.372 (6)
C38	1.0504 (3)	0.2320 (2)	0.05776 (16)	0.0305 (12)	
H38A	1.0861	0.2681	0.0640	0.037*	0.628 (6)
H38B	1.0674	0.1988	0.0384	0.037*	0.372 (6)
C39	1.0385 (3)	0.1962 (2)	0.09990 (16)	0.0276 (12)	
H39A	1.0105	0.2240	0.1203	0.033*	
H39B	1.0018	0.1594	0.0953	0.033*	
C40	1.1217 (4)	0.1743 (3)	0.1179 (2)	0.063 (2)	
H40A	1.1088	0.1437	0.1397	0.075*	0.628 (6)
H40B	1.1469	0.2105	0.1319	0.075*	0.628 (6)
H40C	1.1273	0.1310	0.1080	0.075*	0.372 (6)
H40D	1.1117	0.1712	0.1480	0.075*	0.372 (6)
C41A	1.1863 (5)	0.1466 (5)	0.0918 (3)	0.041 (3)	0.628 (6)
H41A	1.1737	0.1016	0.0876	0.050*	0.628 (6)
H41B	1.2409	0.1497	0.1063	0.050*	0.628 (6)
C42A	1.1928 (6)	0.1786 (5)	0.0491 (3)	0.050 (3)	0.628 (6)
H42A	1.2271	0.1525	0.0306	0.060*	0.628 (6)
H42B	1.2205	0.2192	0.0522	0.060*	0.628 (6)
C43A	1.1051 (5)	0.1885 (4)	0.0290 (3)	0.040 (2)	0.628 (6)
H43A	1.0769	0.1476	0.0254	0.048*	0.628 (6)
H43B	1.1115	0.2080	0.0011	0.048*	0.628 (6)
C41B	1.1983 (9)	0.1995 (7)	0.1156 (4)	0.033 (4)	0.372 (6)
H41C	1.2404	0.1656	0.1175	0.039*	0.372 (6)
H41D	1.2066	0.2277	0.1397	0.039*	0.372 (6)
C42B	1.2129 (7)	0.2360 (6)	0.0756 (4)	0.024 (3)	0.372 (6)
H42C	1.2267	0.2061	0.0530	0.029*	0.372 (6)
H42D	1.2620	0.2637	0.0796	0.029*	0.372 (6)
C43B	1.1385 (8)	0.2757 (7)	0.0619 (5)	0.034 (4)	0.372 (6)
H43C	1.1510	0.2953	0.0345	0.041*	0.372 (6)
H43D	1.1293	0.3096	0.0825	0.041*	0.372 (6)
C44	0.8940 (3)	0.2757 (2)	0.17588 (16)	0.0222 (11)	
C45	0.8187 (3)	0.1704 (2)	0.14532 (16)	0.0249 (11)	
C46	0.6750 (3)	0.2500 (2)	0.12381 (16)	0.0273 (12)	
C47	0.8544 (3)	0.4026 (2)	0.18272 (16)	0.0235 (11)	
C48	0.7813 (3)	0.4784 (2)	0.12459 (15)	0.0227 (11)	
C49	0.6777 (3)	0.3804 (2)	0.09840 (17)	0.0233 (11)	
C50	0.9581 (4)	0.3564 (3)	0.11070 (17)	0.0384 (14)	
C51	0.8451 (4)	0.3972 (2)	0.04620 (18)	0.0370 (14)	
C52	0.7685 (3)	0.2794 (3)	0.05461 (19)	0.0397 (14)	

### Atomic displacement parameters (Å^2^)

**Table:**

	*U*^11^	*U*^22^	*U*^33^	*U*^12^	*U*^13^	*U*^23^
Ru1	0.01512 (19)	0.01609 (18)	0.0211 (2)	0.00056 (15)	0.00138 (16)	−0.00159 (15)
Ru2	0.01401 (19)	0.01614 (18)	0.0196 (2)	−0.00059 (14)	0.00078 (15)	−0.00070 (15)
Ru3	0.0191 (2)	0.02256 (19)	0.0208 (2)	−0.00073 (16)	0.00432 (17)	−0.00093 (17)
As1	0.0128 (2)	0.0144 (2)	0.0210 (3)	0.00013 (18)	0.00052 (19)	−0.00006 (19)
As2	0.0130 (2)	0.0145 (2)	0.0206 (3)	−0.00054 (18)	0.00109 (19)	−0.00064 (19)
P1	0.0264 (8)	0.0408 (8)	0.0260 (8)	0.0088 (6)	0.0061 (6)	0.0046 (6)
O1	0.0181 (19)	0.0258 (18)	0.048 (2)	0.0005 (15)	−0.0017 (17)	0.0056 (17)
O2	0.036 (2)	0.0181 (18)	0.060 (3)	0.0037 (16)	0.0032 (19)	−0.0062 (18)
O3	0.029 (2)	0.048 (2)	0.044 (3)	−0.0117 (18)	−0.0124 (18)	0.0036 (19)
O4	0.028 (2)	0.0334 (19)	0.039 (2)	0.0038 (16)	−0.0118 (18)	−0.0064 (17)
O5	0.036 (2)	0.0230 (18)	0.041 (2)	0.0002 (16)	0.0156 (17)	0.0072 (17)
O6	0.033 (2)	0.047 (2)	0.039 (2)	0.0095 (18)	−0.0193 (19)	−0.0144 (19)
O7	0.035 (3)	0.109 (4)	0.034 (2)	−0.032 (3)	0.002 (2)	−0.005 (2)
O8	0.128 (4)	0.027 (2)	0.050 (3)	0.024 (3)	0.029 (3)	0.011 (2)
O9	0.043 (3)	0.105 (4)	0.048 (3)	−0.031 (3)	0.013 (2)	−0.044 (3)
C1	0.019 (2)	0.015 (2)	0.015 (2)	−0.0028 (18)	−0.0008 (19)	0.0027 (18)
C2	0.050 (4)	0.035 (3)	0.036 (3)	0.021 (3)	−0.016 (3)	−0.008 (3)
C3	0.089 (5)	0.049 (4)	0.034 (4)	0.032 (4)	−0.024 (4)	−0.021 (3)
C4	0.045 (4)	0.043 (3)	0.028 (3)	−0.001 (3)	−0.019 (3)	0.002 (3)
C5	0.020 (3)	0.031 (3)	0.038 (3)	0.001 (2)	−0.010 (2)	0.007 (2)
C6	0.021 (3)	0.028 (3)	0.029 (3)	0.001 (2)	0.001 (2)	−0.003 (2)
C7	0.012 (2)	0.015 (2)	0.033 (3)	0.0018 (18)	−0.004 (2)	0.004 (2)
C8	0.021 (3)	0.017 (2)	0.030 (3)	0.001 (2)	0.006 (2)	0.003 (2)
C9	0.021 (3)	0.024 (3)	0.038 (3)	−0.001 (2)	0.006 (2)	0.010 (2)
C10	0.022 (3)	0.019 (2)	0.050 (4)	−0.006 (2)	−0.001 (3)	0.006 (2)
C11	0.021 (3)	0.021 (2)	0.043 (3)	−0.002 (2)	−0.006 (2)	−0.006 (2)
C12	0.015 (2)	0.021 (2)	0.029 (3)	0.0001 (19)	0.002 (2)	0.002 (2)
C13	0.014 (2)	0.016 (2)	0.024 (3)	−0.0005 (18)	0.0002 (19)	−0.0007 (19)
C14	0.019 (3)	0.015 (2)	0.027 (3)	0.0021 (19)	−0.001 (2)	−0.003 (2)
C15	0.021 (3)	0.029 (3)	0.032 (3)	0.002 (2)	0.004 (2)	0.007 (2)
C16	0.013 (3)	0.045 (3)	0.054 (4)	0.002 (2)	0.006 (3)	0.009 (3)
C17	0.027 (3)	0.031 (3)	0.042 (4)	0.008 (2)	−0.010 (3)	0.001 (3)
C18	0.031 (3)	0.025 (3)	0.029 (3)	0.002 (2)	0.002 (2)	0.006 (2)
C19	0.022 (3)	0.016 (2)	0.033 (3)	0.001 (2)	−0.001 (2)	0.004 (2)
C20	0.015 (2)	0.015 (2)	0.025 (3)	0.0032 (18)	−0.003 (2)	−0.0009 (19)
C21	0.019 (3)	0.024 (2)	0.028 (3)	−0.001 (2)	0.002 (2)	−0.001 (2)
C22	0.020 (3)	0.023 (2)	0.037 (3)	−0.005 (2)	−0.002 (2)	−0.004 (2)
C23	0.022 (3)	0.029 (3)	0.027 (3)	0.001 (2)	0.000 (2)	−0.013 (2)
C24	0.021 (3)	0.034 (3)	0.027 (3)	0.007 (2)	0.007 (2)	−0.002 (2)
C25	0.017 (2)	0.016 (2)	0.030 (3)	0.0016 (19)	0.002 (2)	−0.001 (2)
C26A	0.019 (3)	0.024 (6)	0.030 (3)	0.001 (5)	0.012 (2)	0.009 (5)
C27A	0.018 (5)	0.043 (7)	0.032 (5)	−0.008 (5)	−0.002 (4)	0.006 (5)
C28A	0.034 (6)	0.032 (6)	0.042 (6)	−0.014 (5)	−0.007 (4)	0.017 (6)
C29A	0.036 (7)	0.025 (6)	0.041 (6)	−0.004 (5)	0.006 (5)	0.013 (5)
C30A	0.022 (8)	0.022 (7)	0.021 (5)	0.006 (5)	0.002 (5)	0.001 (5)
C31A	0.011 (5)	0.025 (5)	0.019 (5)	0.004 (4)	0.010 (4)	0.007 (4)
C32A	0.010 (7)	0.034 (5)	0.029 (4)	−0.001 (4)	−0.005 (5)	−0.007 (3)
C33A	0.031 (8)	0.038 (7)	0.031 (6)	0.005 (5)	0.005 (6)	−0.006 (5)
C34A	0.052 (9)	0.046 (8)	0.040 (6)	0.002 (6)	−0.006 (6)	−0.016 (5)
C35A	0.055 (10)	0.051 (7)	0.056 (8)	−0.006 (6)	0.000 (7)	−0.013 (6)
C36A	0.040 (10)	0.042 (7)	0.042 (7)	−0.006 (5)	0.004 (6)	0.004 (5)
C37A	0.042 (11)	0.043 (7)	0.034 (6)	−0.003 (6)	0.002 (6)	0.008 (5)
C26B	0.019 (3)	0.024 (6)	0.030 (3)	0.001 (5)	0.012 (2)	0.009 (5)
C27B	0.024 (9)	0.037 (9)	0.049 (8)	0.013 (7)	0.004 (7)	−0.019 (7)
C28B	0.033 (9)	0.018 (8)	0.076 (10)	−0.012 (8)	−0.001 (7)	−0.005 (9)
C29B	0.025 (10)	0.056 (14)	0.046 (11)	−0.012 (11)	0.016 (8)	−0.011 (9)
C30B	0.018 (10)	0.030 (12)	0.038 (9)	−0.001 (8)	0.008 (7)	0.016 (7)
C31B	0.022 (11)	0.039 (11)	0.039 (11)	−0.006 (8)	0.010 (9)	−0.009 (8)
C32B	0.010 (7)	0.034 (5)	0.029 (4)	−0.001 (4)	−0.005 (5)	−0.007 (3)
C33B	0.017 (10)	0.033 (9)	0.043 (9)	0.000 (7)	−0.009 (7)	−0.012 (7)
C34B	0.035 (12)	0.025 (9)	0.047 (9)	−0.009 (8)	0.002 (9)	−0.016 (6)
C35B	0.069 (16)	0.020 (8)	0.036 (10)	−0.008 (8)	0.004 (11)	−0.010 (7)
C36B	0.032 (14)	0.042 (10)	0.044 (11)	−0.009 (8)	−0.001 (8)	−0.013 (9)
C37B	0.032 (13)	0.032 (10)	0.036 (10)	0.005 (8)	−0.007 (8)	−0.011 (7)
C38	0.025 (3)	0.039 (3)	0.027 (3)	0.006 (2)	−0.001 (2)	0.002 (2)
C39	0.020 (3)	0.037 (3)	0.026 (3)	−0.004 (2)	0.004 (2)	−0.003 (2)
C40	0.042 (4)	0.096 (5)	0.050 (4)	0.007 (4)	0.005 (3)	0.042 (4)
C41A	0.022 (5)	0.059 (6)	0.043 (6)	0.011 (4)	−0.001 (4)	0.025 (5)
C42A	0.037 (6)	0.060 (7)	0.052 (7)	0.004 (5)	0.001 (5)	0.008 (5)
C43A	0.027 (5)	0.059 (6)	0.034 (5)	0.012 (4)	−0.004 (4)	0.007 (5)
C41B	0.037 (9)	0.046 (9)	0.015 (7)	0.015 (7)	0.001 (6)	0.000 (7)
C42B	0.009 (6)	0.025 (7)	0.039 (9)	0.005 (5)	0.007 (6)	0.000 (6)
C43B	0.023 (8)	0.042 (8)	0.037 (9)	−0.010 (6)	0.015 (7)	0.014 (7)
C44	0.015 (3)	0.021 (2)	0.031 (3)	0.0038 (19)	0.000 (2)	0.001 (2)
C45	0.016 (3)	0.029 (3)	0.030 (3)	−0.005 (2)	0.001 (2)	−0.002 (2)
C46	0.026 (3)	0.025 (3)	0.031 (3)	−0.007 (2)	−0.007 (2)	0.004 (2)
C47	0.023 (3)	0.018 (2)	0.029 (3)	0.004 (2)	0.004 (2)	−0.004 (2)
C48	0.020 (3)	0.022 (2)	0.025 (3)	0.007 (2)	0.005 (2)	−0.003 (2)
C49	0.017 (3)	0.019 (2)	0.034 (3)	0.003 (2)	0.001 (2)	−0.006 (2)
C50	0.031 (3)	0.063 (4)	0.021 (3)	−0.010 (3)	0.010 (3)	−0.003 (3)
C51	0.050 (4)	0.027 (3)	0.034 (3)	0.007 (3)	0.010 (3)	−0.004 (3)
C52	0.025 (3)	0.053 (4)	0.042 (4)	−0.006 (3)	0.017 (3)	−0.019 (3)

### Geometric parameters (Å, °)

**Table:**

Ru1—C45	1.897 (5)	C29A—H29B	0.9700
Ru1—C44	1.912 (5)	C30A—C31A	1.480 (10)
Ru1—C46	1.930 (5)	C30A—H30A	0.9700
Ru1—As1	2.4138 (6)	C30A—H30B	0.9700
Ru1—Ru2	2.8699 (5)	C31A—H31A	0.9700
Ru1—Ru3	2.8769 (5)	C31A—H31B	0.9700
Ru2—C48	1.890 (5)	C32A—C33A	1.532 (10)
Ru2—C47	1.920 (5)	C32A—C37A	1.543 (11)
Ru2—C49	1.935 (5)	C32A—H32A	0.9800
Ru2—As2	2.4369 (6)	C33A—C34A	1.526 (12)
Ru2—Ru3	2.8621 (5)	C33A—H33A	0.9700
Ru3—C51	1.866 (6)	C33A—H33B	0.9700
Ru3—C52	1.906 (6)	C34A—C35A	1.536 (13)
Ru3—C50	1.914 (6)	C34A—H34A	0.9700
Ru3—P1	2.3905 (14)	C34A—H34B	0.9700
As1—C1	1.936 (4)	C35A—C36A	1.495 (12)
As1—C7	1.961 (4)	C35A—H35A	0.9700
As1—C13	1.962 (4)	C35A—H35B	0.9700
As2—C20	1.950 (4)	C36A—C37A	1.507 (11)
As2—C14	1.951 (4)	C36A—H36A	0.9700
As2—C13	1.956 (4)	C36A—H36B	0.9700
P1—C32B	1.653 (19)	C37A—H37A	0.9700
P1—C26A	1.801 (16)	C37A—H37B	0.9700
P1—C38	1.844 (5)	C26B—C31B	1.561 (15)
P1—C32A	1.988 (10)	C26B—C27B	1.566 (15)
P1—C26B	2.00 (3)	C26B—H26B	0.9800
O1—C44	1.163 (5)	C27B—C28B	1.468 (15)
O2—C45	1.135 (5)	C27B—H27C	0.9700
O3—C46	1.151 (5)	C27B—H27D	0.9700
O4—C47	1.146 (5)	C28B—C29B	1.508 (15)
O5—C48	1.148 (5)	C28B—H28C	0.9700
O6—C49	1.128 (5)	C28B—H28D	0.9700
O7—C50	1.157 (6)	C29B—C30B	1.557 (16)
O8—C51	1.138 (6)	C29B—H29C	0.9700
O9—C52	1.161 (6)	C29B—H29D	0.9700
C1—C2	1.374 (7)	C30B—C31B	1.461 (15)
C1—C6	1.393 (6)	C30B—H30C	0.9700
C2—C3	1.385 (8)	C30B—H30D	0.9700
C2—H2A	0.9300	C31B—H31C	0.9700
C3—C4	1.375 (7)	C31B—H31D	0.9700
C3—H3A	0.9300	C32B—C33B	1.530 (15)
C4—C5	1.359 (7)	C32B—C37B	1.541 (15)
C4—H4A	0.9300	C32B—H32B	0.9800
C5—C6	1.393 (7)	C33B—C34B	1.531 (15)
C5—H5A	0.9300	C33B—H33C	0.9700
C6—H6A	0.9300	C33B—H33D	0.9700
C7—C8	1.383 (6)	C34B—C35B	1.537 (15)
C7—C12	1.390 (6)	C34B—H34C	0.9700
C8—C9	1.382 (6)	C34B—H34D	0.9700
C8—H8A	0.9300	C35B—C36B	1.508 (15)
C9—C10	1.374 (7)	C35B—H35C	0.9700
C9—H9A	0.9300	C35B—H35D	0.9700
C10—C11	1.379 (7)	C36B—C37B	1.511 (15)
C10—H10A	0.9300	C36B—H36C	0.9700
C11—C12	1.396 (6)	C36B—H36D	0.9700
C11—H11A	0.9300	C37B—H37C	0.9700
C12—H12A	0.9300	C37B—H37D	0.9700
C13—H13A	0.9700	C38—C39	1.530 (7)
C13—H13B	0.9700	C38—C43A	1.540 (10)
C14—C19	1.383 (6)	C38—C43B	1.660 (13)
C14—C15	1.393 (6)	C38—H38A	0.9599
C15—C16	1.382 (6)	C38—H38B	0.9599
C15—H15A	0.9300	C39—C40	1.491 (7)
C16—C17	1.379 (7)	C39—H39A	0.9700
C16—H16A	0.9300	C39—H39B	0.9700
C17—C18	1.373 (7)	C40—C41B	1.311 (15)
C17—H17A	0.9300	C40—C41A	1.424 (11)
C18—C19	1.379 (6)	C40—H40A	0.9601
C18—H18A	0.9300	C40—H40B	0.9601
C19—H19A	0.9300	C40—H40C	0.9599
C20—C25	1.390 (6)	C40—H40D	0.9601
C20—C21	1.404 (6)	C41A—C42A	1.501 (12)
C21—C22	1.388 (6)	C41A—H40C	1.1027
C21—H21A	0.9300	C41A—H41A	0.9700
C22—C23	1.378 (7)	C41A—H41B	0.9700
C22—H22A	0.9300	C42A—C43A	1.523 (12)
C23—C24	1.374 (6)	C42A—H42A	0.9601
C23—H23A	0.9300	C42A—H42B	0.9600
C24—C25	1.394 (6)	C43A—H38B	0.6930
C24—H24A	0.9300	C43A—H43A	0.9700
C25—H25A	0.9300	C43A—H43B	0.9700
C26A—C27A	1.559 (10)	C41B—C42B	1.485 (18)
C26A—C31A	1.561 (11)	C41B—H40B	0.9794
C26A—H26A	0.9800	C41B—H41C	0.9700
C27A—C28A	1.486 (10)	C41B—H41D	0.9700
C27A—H27A	0.9700	C42B—C43B	1.494 (17)
C27A—H27B	0.9700	C42B—H42B	0.8214
C28A—C29A	1.509 (11)	C42B—H42C	0.9700
C28A—H28A	0.9700	C42B—H42D	0.9700
C28A—H28B	0.9700	C43B—H38A	0.8380
C29A—C30A	1.554 (10)	C43B—H43C	0.9700
C29A—H29A	0.9700	C43B—H43D	0.9700
			
C45—Ru1—C44	89.21 (19)	C33A—C34A—C35A	112.2 (9)
C45—Ru1—C46	97.6 (2)	C33A—C34A—H34A	109.2
C44—Ru1—C46	172.98 (19)	C35A—C34A—H34A	109.2
C45—Ru1—As1	100.47 (15)	C33A—C34A—H34B	109.2
C44—Ru1—As1	95.42 (15)	C35A—C34A—H34B	109.2
C46—Ru1—As1	85.09 (15)	H34A—C34A—H34B	107.9
C45—Ru1—Ru2	166.63 (15)	C36A—C35A—C34A	108.9 (9)
C44—Ru1—Ru2	86.96 (13)	C36A—C35A—H35A	109.9
C46—Ru1—Ru2	86.03 (14)	C34A—C35A—H35A	109.9
As1—Ru1—Ru2	92.653 (17)	C36A—C35A—H35B	109.9
C45—Ru1—Ru3	107.03 (15)	C34A—C35A—H35B	109.9
C44—Ru1—Ru3	81.86 (14)	H35A—C35A—H35B	108.3
C46—Ru1—Ru3	94.42 (15)	C35A—C36A—C37A	113.7 (11)
As1—Ru1—Ru3	152.304 (19)	C35A—C36A—H36A	108.8
Ru2—Ru1—Ru3	59.741 (12)	C37A—C36A—H36A	108.8
C48—Ru2—C47	89.1 (2)	C35A—C36A—H36B	108.8
C48—Ru2—C49	91.4 (2)	C37A—C36A—H36B	108.8
C47—Ru2—C49	176.01 (19)	H36A—C36A—H36B	107.7
C48—Ru2—As2	103.99 (14)	C36A—C37A—C32A	111.8 (9)
C47—Ru2—As2	92.68 (14)	C36A—C37A—H37A	109.3
C49—Ru2—As2	91.05 (14)	C32A—C37A—H37A	109.3
C48—Ru2—Ru3	101.32 (14)	C36A—C37A—H37B	109.3
C47—Ru2—Ru3	96.26 (13)	C32A—C37A—H37B	109.3
C49—Ru2—Ru3	79.76 (13)	H37A—C37A—H37B	107.9
As2—Ru2—Ru3	153.251 (19)	C31B—C26B—C27B	109.4 (15)
C48—Ru2—Ru1	160.66 (13)	C31B—C26B—P1	114.7 (17)
C47—Ru2—Ru1	87.40 (13)	C27B—C26B—P1	117.1 (18)
C49—Ru2—Ru1	90.85 (13)	C31B—C26B—H26B	104.7
As2—Ru2—Ru1	95.171 (17)	C27B—C26B—H26B	104.7
Ru3—Ru2—Ru1	60.251 (13)	P1—C26B—H26B	104.7
C51—Ru3—C52	93.7 (3)	C28B—C27B—C26B	111.1 (14)
C51—Ru3—C50	96.4 (2)	C28B—C27B—H27C	109.4
C52—Ru3—C50	169.9 (3)	C26B—C27B—H27C	109.4
C51—Ru3—P1	99.51 (16)	C28B—C27B—H27D	109.4
C52—Ru3—P1	86.64 (16)	C26B—C27B—H27D	109.4
C50—Ru3—P1	91.13 (17)	H27C—C27B—H27D	108.0
C51—Ru3—Ru2	84.57 (16)	C27B—C28B—C29B	115.3 (15)
C52—Ru3—Ru2	97.00 (15)	C27B—C28B—H28C	108.4
C50—Ru3—Ru2	84.55 (16)	C29B—C28B—H28C	108.4
P1—Ru3—Ru2	174.39 (4)	C27B—C28B—H28D	108.4
C51—Ru3—Ru1	140.89 (16)	C29B—C28B—H28D	108.4
C52—Ru3—Ru1	76.23 (18)	H28C—C28B—H28D	107.5
C50—Ru3—Ru1	96.13 (16)	C28B—C29B—C30B	108.6 (16)
P1—Ru3—Ru1	117.11 (4)	C28B—C29B—H29C	110.0
Ru2—Ru3—Ru1	60.008 (12)	C30B—C29B—H29C	110.0
C1—As1—C7	102.29 (18)	C28B—C29B—H29D	110.0
C1—As1—C13	106.47 (19)	C30B—C29B—H29D	110.0
C7—As1—C13	96.59 (17)	H29C—C29B—H29D	108.4
C1—As1—Ru1	117.05 (13)	C31B—C30B—C29B	113.5 (18)
C7—As1—Ru1	118.18 (14)	C31B—C30B—H30C	108.9
C13—As1—Ru1	113.68 (13)	C29B—C30B—H30C	108.9
C20—As2—C14	98.75 (18)	C31B—C30B—H30D	108.9
C20—As2—C13	103.96 (18)	C29B—C30B—H30D	108.9
C14—As2—C13	102.23 (18)	H30C—C30B—H30D	107.7
C20—As2—Ru2	120.55 (13)	C30B—C31B—C26B	116.8 (16)
C14—As2—Ru2	114.26 (14)	C30B—C31B—H31C	108.1
C13—As2—Ru2	114.48 (13)	C26B—C31B—H31C	108.1
C32B—P1—C26A	96.3 (11)	C30B—C31B—H31D	108.1
C32B—P1—C38	102.5 (11)	C26B—C31B—H31D	108.1
C26A—P1—C38	102.4 (6)	H31C—C31B—H31D	107.3
C26A—P1—C32A	99.7 (6)	C33B—C32B—C37B	107.7 (15)
C38—P1—C32A	107.9 (5)	C33B—C32B—P1	119.6 (14)
C32B—P1—C26B	105.1 (12)	C37B—C32B—P1	117.1 (18)
C38—P1—C26B	103.7 (10)	C33B—C32B—H32B	103.3
C32A—P1—C26B	107.9 (9)	C37B—C32B—H32B	103.3
C32B—P1—Ru3	120.6 (8)	P1—C32B—H32B	103.3
C26A—P1—Ru3	115.8 (5)	C32B—C33B—C34B	111.6 (13)
C38—P1—Ru3	115.98 (18)	C32B—C33B—H33C	109.3
C32A—P1—Ru3	113.4 (4)	C34B—C33B—H33C	109.3
C26B—P1—Ru3	107.3 (10)	C32B—C33B—H33D	109.3
C2—C1—C6	118.7 (4)	C34B—C33B—H33D	109.3
C2—C1—As1	124.2 (4)	H33C—C33B—H33D	108.0
C6—C1—As1	117.1 (3)	C33B—C34B—C35B	112.9 (13)
C1—C2—C3	120.5 (5)	C33B—C34B—H34C	109.0
C1—C2—H2A	119.8	C35B—C34B—H34C	109.0
C3—C2—H2A	119.8	C33B—C34B—H34D	109.0
C4—C3—C2	120.6 (5)	C35B—C34B—H34D	109.0
C4—C3—H3A	119.7	H34C—C34B—H34D	107.8
C2—C3—H3A	119.7	C36B—C35B—C34B	108.0 (13)
C5—C4—C3	119.6 (5)	C36B—C35B—H35C	110.1
C5—C4—H4A	120.2	C34B—C35B—H35C	110.1
C3—C4—H4A	120.2	C36B—C35B—H35D	110.1
C4—C5—C6	120.5 (5)	C34B—C35B—H35D	110.1
C4—C5—H5A	119.8	H35C—C35B—H35D	108.4
C6—C5—H5A	119.8	C35B—C36B—C37B	112.2 (17)
C1—C6—C5	120.1 (5)	C35B—C36B—H36C	109.2
C1—C6—H6A	119.9	C37B—C36B—H36C	109.2
C5—C6—H6A	119.9	C35B—C36B—H36D	109.2
C8—C7—C12	120.5 (4)	C37B—C36B—H36D	109.2
C8—C7—As1	119.3 (3)	H36C—C36B—H36D	107.9
C12—C7—As1	120.1 (4)	C36B—C37B—C32B	112.3 (15)
C9—C8—C7	119.3 (5)	C36B—C37B—H37C	109.1
C9—C8—H8A	120.4	C32B—C37B—H37C	109.1
C7—C8—H8A	120.4	C36B—C37B—H37D	109.1
C10—C9—C8	121.0 (5)	C32B—C37B—H37D	109.1
C10—C9—H9A	119.5	H37C—C37B—H37D	107.9
C8—C9—H9A	119.5	C39—C38—C43A	106.5 (5)
C9—C10—C11	119.9 (4)	C39—C38—C43B	107.7 (6)
C9—C10—H10A	120.0	C43A—C38—C43B	84.8 (7)
C11—C10—H10A	120.0	C39—C38—P1	115.9 (3)
C10—C11—C12	120.1 (5)	C43A—C38—P1	116.0 (4)
C10—C11—H11A	119.9	C43B—C38—P1	121.2 (6)
C12—C11—H11A	119.9	C39—C38—H38A	106.2
C7—C12—C11	119.1 (5)	C43A—C38—H38A	105.1
C7—C12—H12A	120.4	P1—C38—H38A	106.1
C11—C12—H12A	120.4	C39—C38—H38B	103.1
As2—C13—As1	112.2 (2)	C43B—C38—H38B	102.5
As2—C13—H13A	109.2	P1—C38—H38B	103.7
As1—C13—H13A	109.2	H38A—C38—H38B	122.4
As2—C13—H13B	109.2	C40—C39—C38	111.7 (4)
As1—C13—H13B	109.2	C40—C39—H39A	109.3
H13A—C13—H13B	107.9	C38—C39—H39A	109.3
C19—C14—C15	119.5 (4)	C40—C39—H39B	109.3
C19—C14—As2	118.2 (3)	C38—C39—H39B	109.3
C15—C14—As2	122.1 (4)	H39A—C39—H39B	107.9
C16—C15—C14	119.7 (5)	C41B—C40—C41A	58.9 (7)
C16—C15—H15A	120.2	C41B—C40—C39	130.9 (8)
C14—C15—H15A	120.2	C41A—C40—C39	121.9 (6)
C17—C16—C15	120.1 (5)	C41B—C40—H40A	120.0
C17—C16—H16A	119.9	C41A—C40—H40A	106.7
C15—C16—H16A	119.9	C39—C40—H40A	106.9
C18—C17—C16	120.3 (5)	C41B—C40—H40B	48.1
C18—C17—H17A	119.9	C41A—C40—H40B	107.0
C16—C17—H17A	119.9	C39—C40—H40B	106.8
C17—C18—C19	120.0 (5)	H40A—C40—H40B	106.6
C17—C18—H18A	120.0	C41B—C40—H40C	106.1
C19—C18—H18A	120.0	C41A—C40—H40C	50.7
C18—C19—C14	120.3 (4)	C39—C40—H40C	104.4
C18—C19—H19A	119.8	H40A—C40—H40C	67.7
C14—C19—H19A	119.8	H40B—C40—H40C	148.5
C25—C20—C21	119.5 (4)	C41B—C40—H40D	103.3
C25—C20—As2	122.5 (3)	C41A—C40—H40D	130.9
C21—C20—As2	117.7 (3)	C39—C40—H40D	104.5
C22—C21—C20	119.4 (4)	H40B—C40—H40D	70.7
C22—C21—H21A	120.3	H40C—C40—H40D	105.6
C20—C21—H21A	120.3	C40—C41A—C42A	112.2 (7)
C23—C22—C21	120.6 (4)	C42A—C41A—H40C	126.9
C23—C22—H22A	119.7	C40—C41A—H41A	109.2
C21—C22—H22A	119.7	C42A—C41A—H41A	109.2
C24—C23—C22	120.3 (4)	H40C—C41A—H41A	66.8
C24—C23—H23A	119.9	C40—C41A—H41B	109.2
C22—C23—H23A	119.9	C42A—C41A—H41B	109.2
C23—C24—C25	120.2 (4)	H40C—C41A—H41B	122.7
C23—C24—H24A	119.9	H41A—C41A—H41B	107.9
C25—C24—H24A	119.9	C41A—C42A—C43A	111.6 (8)
C20—C25—C24	120.0 (4)	C41A—C42A—H42A	108.8
C20—C25—H25A	120.0	C43A—C42A—H42A	109.4
C24—C25—H25A	120.0	C41A—C42A—H42B	109.6
C27A—C26A—C31A	109.4 (8)	C43A—C42A—H42B	109.2
C27A—C26A—P1	111.4 (10)	H42A—C42A—H42B	108.2
C31A—C26A—P1	112.0 (11)	C42A—C43A—C38	109.8 (7)
C27A—C26A—H26A	108.0	C42A—C43A—H38B	129.2
C31A—C26A—H26A	108.0	C42A—C43A—H43A	109.7
P1—C26A—H26A	108.0	C38—C43A—H43A	109.7
C28A—C27A—C26A	111.5 (8)	H38B—C43A—H43A	86.4
C28A—C27A—H27A	109.3	C42A—C43A—H43B	109.7
C26A—C27A—H27A	109.3	C38—C43A—H43B	109.7
C28A—C27A—H27B	109.3	H38B—C43A—H43B	109.9
C26A—C27A—H27B	109.3	H43A—C43A—H43B	108.2
H27A—C27A—H27B	108.0	C40—C41B—C42B	113.2 (11)
C27A—C28A—C29A	113.8 (9)	C40—C41B—H40B	46.9
C27A—C28A—H28A	108.8	C42B—C41B—H40B	116.4
C29A—C28A—H28A	108.8	C40—C41B—H41C	108.9
C27A—C28A—H28B	108.8	C42B—C41B—H41C	108.9
C29A—C28A—H28B	108.8	H40B—C41B—H41C	134.1
H28A—C28A—H28B	107.7	C40—C41B—H41D	108.9
C28A—C29A—C30A	109.7 (9)	C42B—C41B—H41D	108.9
C28A—C29A—H29A	109.7	H40B—C41B—H41D	63.8
C30A—C29A—H29A	109.7	H41C—C41B—H41D	107.8
C28A—C29A—H29B	109.7	C41B—C42B—C43B	114.1 (11)
C30A—C29A—H29B	109.7	C41B—C42B—H42B	123.8
H29A—C29A—H29B	108.2	C43B—C42B—H42B	95.3
C31A—C30A—C29A	111.9 (9)	C41B—C42B—H42C	108.7
C31A—C30A—H30A	109.2	C43B—C42B—H42C	108.7
C29A—C30A—H30A	109.2	C41B—C42B—H42D	108.7
C31A—C30A—H30B	109.2	C43B—C42B—H42D	108.7
C29A—C30A—H30B	109.2	H42B—C42B—H42D	104.8
H30A—C30A—H30B	107.9	H42C—C42B—H42D	107.6
C30A—C31A—C26A	112.9 (11)	C42B—C43B—C38	111.3 (10)
C30A—C31A—H31A	109.0	C42B—C43B—H38A	129.2
C26A—C31A—H31A	109.0	C42B—C43B—H43C	109.4
C30A—C31A—H31B	109.0	C38—C43B—H43C	109.4
C26A—C31A—H31B	109.0	H38A—C43B—H43C	110.5
H31A—C31A—H31B	107.8	C42B—C43B—H43D	109.4
C33A—C32A—C37A	108.9 (9)	C38—C43B—H43D	109.4
C33A—C32A—P1	118.2 (7)	H38A—C43B—H43D	86.6
C37A—C32A—P1	113.0 (10)	H43C—C43B—H43D	108.0
C33A—C32A—H32A	105.2	O1—C44—Ru1	173.2 (4)
C37A—C32A—H32A	105.2	O2—C45—Ru1	176.3 (4)
P1—C32A—H32A	105.2	O3—C46—Ru1	173.0 (5)
C34A—C33A—C32A	110.7 (8)	O4—C47—Ru2	173.1 (4)
C34A—C33A—H33A	109.5	O5—C48—Ru2	177.7 (4)
C32A—C33A—H33A	109.5	O6—C49—Ru2	174.5 (4)
C34A—C33A—H33B	109.5	O7—C50—Ru3	174.4 (5)
C32A—C33A—H33B	109.5	O8—C51—Ru3	174.5 (5)
H33A—C33A—H33B	108.1	O9—C52—Ru3	172.6 (5)
			
C45—Ru1—Ru2—C48	−10.2 (8)	As1—C7—C12—C11	−177.5 (3)
C44—Ru1—Ru2—C48	63.3 (5)	C10—C11—C12—C7	0.0 (7)
C46—Ru1—Ru2—C48	−116.5 (5)	C20—As2—C13—As1	−107.7 (2)
As1—Ru1—Ru2—C48	158.6 (4)	C14—As2—C13—As1	149.9 (2)
Ru3—Ru1—Ru2—C48	−19.0 (4)	Ru2—As2—C13—As1	25.8 (3)
C45—Ru1—Ru2—C47	−89.9 (6)	C1—As1—C13—As2	91.1 (2)
C44—Ru1—Ru2—C47	−16.4 (2)	C7—As1—C13—As2	−164.0 (2)
C46—Ru1—Ru2—C47	163.8 (2)	Ru1—As1—C13—As2	−39.2 (2)
As1—Ru1—Ru2—C47	78.91 (14)	C20—As2—C14—C19	95.6 (4)
Ru3—Ru1—Ru2—C47	−98.73 (14)	C13—As2—C14—C19	−158.0 (4)
C45—Ru1—Ru2—C49	86.5 (6)	Ru2—As2—C14—C19	−33.7 (4)
C44—Ru1—Ru2—C49	160.0 (2)	C20—As2—C14—C15	−80.3 (4)
C46—Ru1—Ru2—C49	−19.8 (2)	C13—As2—C14—C15	26.1 (4)
As1—Ru1—Ru2—C49	−104.67 (14)	Ru2—As2—C14—C15	150.4 (3)
Ru3—Ru1—Ru2—C49	77.69 (14)	C19—C14—C15—C16	0.0 (7)
C45—Ru1—Ru2—As2	177.6 (6)	As2—C14—C15—C16	175.8 (4)
C44—Ru1—Ru2—As2	−108.84 (15)	C14—C15—C16—C17	1.1 (8)
C46—Ru1—Ru2—As2	71.35 (16)	C15—C16—C17—C18	−0.8 (8)
As1—Ru1—Ru2—As2	−13.54 (2)	C16—C17—C18—C19	−0.5 (8)
Ru3—Ru1—Ru2—As2	168.818 (19)	C17—C18—C19—C14	1.5 (7)
C45—Ru1—Ru2—Ru3	8.8 (6)	C15—C14—C19—C18	−1.3 (7)
C44—Ru1—Ru2—Ru3	82.35 (15)	As2—C14—C19—C18	−177.3 (4)
C46—Ru1—Ru2—Ru3	−97.47 (16)	C14—As2—C20—C25	76.4 (4)
As1—Ru1—Ru2—Ru3	177.639 (19)	C13—As2—C20—C25	−28.6 (4)
C48—Ru2—Ru3—C51	−23.6 (2)	Ru2—As2—C20—C25	−158.6 (3)
C47—Ru2—Ru3—C51	−113.9 (2)	C14—As2—C20—C21	−97.1 (4)
C49—Ru2—Ru3—C51	65.8 (2)	C13—As2—C20—C21	157.8 (3)
As2—Ru2—Ru3—C51	137.30 (18)	Ru2—As2—C20—C21	27.9 (4)
Ru1—Ru2—Ru3—C51	162.72 (18)	C25—C20—C21—C22	0.4 (6)
C48—Ru2—Ru3—C52	−116.7 (2)	As2—C20—C21—C22	174.2 (3)
C47—Ru2—Ru3—C52	153.0 (2)	C20—C21—C22—C23	0.0 (7)
C49—Ru2—Ru3—C52	−27.3 (2)	C21—C22—C23—C24	0.2 (7)
As2—Ru2—Ru3—C52	44.2 (2)	C22—C23—C24—C25	−0.9 (7)
Ru1—Ru2—Ru3—C52	69.65 (19)	C21—C20—C25—C24	−1.1 (6)
C48—Ru2—Ru3—C50	73.4 (2)	As2—C20—C25—C24	−174.5 (3)
C47—Ru2—Ru3—C50	−16.9 (2)	C23—C24—C25—C20	1.3 (7)
C49—Ru2—Ru3—C50	162.8 (2)	C32B—P1—C26A—C27A	−170.5 (14)
As2—Ru2—Ru3—C50	−125.72 (18)	C38—P1—C26A—C27A	−66.2 (11)
Ru1—Ru2—Ru3—C50	−100.31 (17)	C32A—P1—C26A—C27A	−177.0 (10)
C48—Ru2—Ru3—Ru1	173.68 (15)	C26B—P1—C26A—C27A	32 (8)
C47—Ru2—Ru3—Ru1	83.38 (14)	Ru3—P1—C26A—C27A	61.0 (11)
C49—Ru2—Ru3—Ru1	−96.93 (14)	C32B—P1—C26A—C31A	66.7 (15)
As2—Ru2—Ru3—Ru1	−25.41 (4)	C38—P1—C26A—C31A	171.0 (10)
C45—Ru1—Ru3—C51	154.2 (3)	C32A—P1—C26A—C31A	60.1 (11)
C44—Ru1—Ru3—C51	−119.2 (3)	C26B—P1—C26A—C31A	−90 (9)
C46—Ru1—Ru3—C51	54.8 (3)	Ru3—P1—C26A—C31A	−61.8 (11)
As1—Ru1—Ru3—C51	−33.0 (3)	C31A—C26A—C27A—C28A	−52.6 (16)
Ru2—Ru1—Ru3—C51	−28.0 (3)	P1—C26A—C27A—C28A	−176.9 (9)
C45—Ru1—Ru3—C52	75.5 (2)	C26A—C27A—C28A—C29A	56.1 (14)
C44—Ru1—Ru3—C52	162.1 (2)	C27A—C28A—C29A—C30A	−55.5 (17)
C46—Ru1—Ru3—C52	−23.9 (2)	C28A—C29A—C30A—C31A	54.5 (19)
As1—Ru1—Ru3—C52	−111.72 (17)	C29A—C30A—C31A—C26A	−55.2 (18)
Ru2—Ru1—Ru3—C52	−106.64 (17)	C27A—C26A—C31A—C30A	53.5 (17)
C45—Ru1—Ru3—C50	−97.8 (2)	P1—C26A—C31A—C30A	177.5 (11)
C44—Ru1—Ru3—C50	−11.1 (2)	C32B—P1—C32A—C33A	−23 (11)
C46—Ru1—Ru3—C50	162.9 (2)	C26A—P1—C32A—C33A	40.4 (12)
As1—Ru1—Ru3—C50	75.00 (18)	C38—P1—C32A—C33A	−66.1 (11)
Ru2—Ru1—Ru3—C50	80.08 (18)	C26B—P1—C32A—C33A	45.3 (16)
C45—Ru1—Ru3—P1	−3.37 (16)	Ru3—P1—C32A—C33A	164.0 (9)
C44—Ru1—Ru3—P1	83.29 (14)	C32B—P1—C32A—C37A	106 (12)
C46—Ru1—Ru3—P1	−102.70 (15)	C26A—P1—C32A—C37A	169.2 (13)
As1—Ru1—Ru3—P1	169.44 (5)	C38—P1—C32A—C37A	62.7 (12)
Ru2—Ru1—Ru3—P1	174.52 (4)	C26B—P1—C32A—C37A	174.1 (16)
C45—Ru1—Ru3—Ru2	−177.88 (15)	Ru3—P1—C32A—C37A	−67.1 (12)
C44—Ru1—Ru3—Ru2	−91.23 (13)	C37A—C32A—C33A—C34A	56.0 (14)
C46—Ru1—Ru3—Ru2	82.79 (15)	P1—C32A—C33A—C34A	−173.2 (10)
As1—Ru1—Ru3—Ru2	−5.08 (4)	C32A—C33A—C34A—C35A	−58.0 (14)
C45—Ru1—As1—C1	83.3 (2)	C33A—C34A—C35A—C36A	55.6 (15)
C44—Ru1—As1—C1	−6.90 (19)	C34A—C35A—C36A—C37A	−54.7 (18)
C46—Ru1—As1—C1	−179.88 (19)	C35A—C36A—C37A—C32A	56 (2)
Ru2—Ru1—As1—C1	−94.10 (14)	C33A—C32A—C37A—C36A	−55.0 (17)
Ru3—Ru1—As1—C1	−89.71 (14)	P1—C32A—C37A—C36A	171.4 (13)
C45—Ru1—As1—C7	−39.7 (2)	C32B—P1—C26B—C31B	47 (3)
C44—Ru1—As1—C7	−129.91 (19)	C26A—P1—C26B—C31B	70 (8)
C46—Ru1—As1—C7	57.1 (2)	C38—P1—C26B—C31B	154 (2)
Ru2—Ru1—As1—C7	142.90 (14)	C32A—P1—C26B—C31B	40 (2)
Ru3—Ru1—As1—C7	147.29 (14)	Ru3—P1—C26B—C31B	−83 (2)
C45—Ru1—As1—C13	−151.9 (2)	C32B—P1—C26B—C27B	177 (2)
C44—Ru1—As1—C13	117.94 (19)	C26A—P1—C26B—C27B	−159 (10)
C46—Ru1—As1—C13	−55.03 (19)	C38—P1—C26B—C27B	−76 (2)
Ru2—Ru1—As1—C13	30.75 (14)	C32A—P1—C26B—C27B	170.2 (17)
Ru3—Ru1—As1—C13	35.14 (14)	Ru3—P1—C26B—C27B	48 (2)
C48—Ru2—As2—C20	−56.2 (2)	C31B—C26B—C27B—C28B	−49 (3)
C47—Ru2—As2—C20	33.48 (19)	P1—C26B—C27B—C28B	178.0 (17)
C49—Ru2—As2—C20	−147.95 (19)	C26B—C27B—C28B—C29B	59 (2)
Ru3—Ru2—As2—C20	143.07 (14)	C27B—C28B—C29B—C30B	−57 (3)
Ru1—Ru2—As2—C20	121.11 (14)	C28B—C29B—C30B—C31B	50 (4)
C48—Ru2—As2—C14	61.1 (2)	C29B—C30B—C31B—C26B	−48 (4)
C47—Ru2—As2—C14	150.85 (19)	C27B—C26B—C31B—C30B	47 (4)
C49—Ru2—As2—C14	−30.57 (19)	P1—C26B—C31B—C30B	−180 (3)
Ru3—Ru2—As2—C14	−99.56 (14)	C26A—P1—C32B—C33B	39 (2)
Ru1—Ru2—As2—C14	−121.52 (14)	C38—P1—C32B—C33B	−65 (2)
C48—Ru2—As2—C13	178.6 (2)	C32A—P1—C32B—C33B	157 (14)
C47—Ru2—As2—C13	−91.73 (19)	C26B—P1—C32B—C33B	43 (2)
C49—Ru2—As2—C13	86.8 (2)	Ru3—P1—C32B—C33B	164.3 (16)
Ru3—Ru2—As2—C13	17.87 (16)	C26A—P1—C32B—C37B	172 (2)
Ru1—Ru2—As2—C13	−4.10 (15)	C38—P1—C32B—C37B	68 (2)
C51—Ru3—P1—C32B	−104.8 (13)	C32A—P1—C32B—C37B	−70 (12)
C52—Ru3—P1—C32B	−11.6 (13)	C26B—P1—C32B—C37B	176 (2)
C50—Ru3—P1—C32B	158.5 (13)	Ru3—P1—C32B—C37B	−62 (2)
Ru1—Ru3—P1—C32B	61.0 (13)	C37B—C32B—C33B—C34B	55 (2)
C51—Ru3—P1—C26A	10.5 (5)	P1—C32B—C33B—C34B	−168 (2)
C52—Ru3—P1—C26A	103.7 (5)	C32B—C33B—C34B—C35B	−57 (2)
C50—Ru3—P1—C26A	−86.1 (5)	C33B—C34B—C35B—C36B	55 (2)
Ru1—Ru3—P1—C26A	176.4 (5)	C34B—C35B—C36B—C37B	−56 (3)
C51—Ru3—P1—C38	130.6 (3)	C35B—C36B—C37B—C32B	60 (3)
C52—Ru3—P1—C38	−136.2 (3)	C33B—C32B—C37B—C36B	−57 (3)
C50—Ru3—P1—C38	33.9 (3)	P1—C32B—C37B—C36B	165 (2)
Ru1—Ru3—P1—C38	−63.6 (2)	C32B—P1—C38—C39	−84.6 (10)
C51—Ru3—P1—C32A	−103.8 (6)	C26A—P1—C38—C39	175.9 (6)
C52—Ru3—P1—C32A	−10.6 (6)	C32A—P1—C38—C39	−79.5 (6)
C50—Ru3—P1—C32A	159.5 (6)	C26B—P1—C38—C39	166.2 (9)
Ru1—Ru3—P1—C32A	62.1 (5)	Ru3—P1—C38—C39	48.8 (4)
C51—Ru3—P1—C26B	15.3 (8)	C32B—P1—C38—C43A	41.5 (10)
C52—Ru3—P1—C26B	108.5 (8)	C26A—P1—C38—C43A	−58.0 (7)
C50—Ru3—P1—C26B	−81.4 (8)	C32A—P1—C38—C43A	46.6 (7)
Ru1—Ru3—P1—C26B	−178.8 (8)	C26B—P1—C38—C43A	−67.7 (10)
C7—As1—C1—C2	−100.0 (4)	Ru3—P1—C38—C43A	174.9 (4)
C13—As1—C1—C2	0.8 (5)	C32B—P1—C38—C43B	141.7 (11)
Ru1—As1—C1—C2	129.2 (4)	C26A—P1—C38—C43B	42.3 (8)
C7—As1—C1—C6	80.1 (4)	C32A—P1—C38—C43B	146.8 (8)
C13—As1—C1—C6	−179.1 (3)	C26B—P1—C38—C43B	32.6 (11)
Ru1—As1—C1—C6	−50.7 (4)	Ru3—P1—C38—C43B	−84.8 (7)
C6—C1—C2—C3	0.6 (8)	C43A—C38—C39—C40	51.8 (7)
As1—C1—C2—C3	−179.3 (5)	C43B—C38—C39—C40	−37.9 (8)
C1—C2—C3—C4	−0.1 (10)	P1—C38—C39—C40	−177.4 (4)
C2—C3—C4—C5	−0.8 (10)	C38—C39—C40—C41B	31.7 (13)
C3—C4—C5—C6	1.1 (8)	C38—C39—C40—C41A	−42.8 (9)
C2—C1—C6—C5	−0.3 (7)	C41B—C40—C41A—C42A	−83.1 (10)
As1—C1—C6—C5	179.6 (4)	C39—C40—C41A—C42A	38.5 (12)
C4—C5—C6—C1	−0.5 (7)	C40—C41A—C42A—C43A	−46.1 (12)
C1—As1—C7—C8	50.2 (4)	C41A—C42A—C43A—C38	61.2 (10)
C13—As1—C7—C8	−58.3 (4)	C39—C38—C43A—C42A	−63.0 (8)
Ru1—As1—C7—C8	−179.6 (3)	C43B—C38—C43A—C42A	44.0 (8)
C1—As1—C7—C12	−132.6 (4)	P1—C38—C43A—C42A	166.3 (6)
C13—As1—C7—C12	118.9 (4)	C41A—C40—C41B—C42B	76.7 (11)
Ru1—As1—C7—C12	−2.4 (4)	C39—C40—C41B—C42B	−30.4 (18)
C12—C7—C8—C9	0.3 (7)	C40—C41B—C42B—C43B	40.9 (17)
As1—C7—C8—C9	177.5 (3)	C41B—C42B—C43B—C38	−54.4 (15)
C7—C8—C9—C10	−0.1 (7)	C39—C38—C43B—C42B	52.8 (12)
C8—C9—C10—C11	−0.2 (7)	C43A—C38—C43B—C42B	−52.9 (11)
C9—C10—C11—C12	0.2 (7)	P1—C38—C43B—C42B	−170.3 (8)
C8—C7—C12—C11	−0.3 (7)		

### Hydrogen-bond geometry (Å, °)

**Table:**

*D*—H···*A*	*D*—H	H···*A*	*D*···*A*	*D*—H···*A*
C31A—H31B···O8	0.97	2.56	3.481 (17)	159
C32A—H32A···O9	0.98	2.47	3.157 (14)	127
C43A—H43B···O9^i^	0.97	2.18	2.954 (10)	135
C5—H5A···Cg1^ii^	0.93	2.94	3.728 (5)	144
C10—H10A···Cg2^iii^	0.93	2.90	3.651 (5)	139
C16—H16A···Cg2^iv^	0.93	2.96	3.718 (5)	140
C22—H22A···Cg1^v^	0.93	2.82	3.608 (5)	143
C41B—H41D···Cg3^ii^	0.97	2.62	3.420 (14)	140

Symmetry codes: (i) *x*+1/2, −*y*+1/2, −*z*; (ii) *x*−1/2, *y*, −*z*−1/2; (iii) *x*+3/2, −*y*−1/2, −*z*; (iv) *x*−3/2, *y*, −*z*−1/2; (v) *x*+1, −*y*−1/2, *z*−1/2.

## References

[bb1] Bruce, M. I., Liddell, M. J., Hughes, C. A., Patrick, J. M., Skelton, B. W. & White, A. H. (1988*a*). *J. Organomet. Chem.* **347**, 181–205.

[bb2] Bruce, M. I., Liddell, M. J., Shawkataly, O. bin, Hughes, C. A., Skelton, B. W. & White, A. H. (1988*b*). *J. Organomet. Chem.* **347**, 207–235.

[bb3] Bruce, M. I., Matisons, J. G. & Nicholson, B. K. (1983). *J. Organomet. Chem.* **247**, 321–343.

[bb4] Bruce, M. I., Shawkataly, O. bin & Williams, M. L. (1985). *J. Organomet. Chem.* **287**, 127–131.

[bb5] Bruker (2005). *APEX2*, *SAINT* and *SADABS*. Bruker AXS Inc., Madison, Wisconsin, USA.

[bb6] Cosier, J. & Glazer, A. M. (1986). *J. Appl. Cryst.* **19**, 105–107.

[bb7] Cremer, D. & Pople, J. A. (1975). *J. Am. Chem. Soc.* **97**, 1354–1358.

[bb8] Shawkataly, O. bin, Khan, I. A., Yeap, C. S. & Fun, H.-K. (2009*a*). *Acta Cryst.* E**65**, m1622–m1623. 10.1107/S1600536809046704 PMC2972049 21578644

[bb9] Shawkataly, O. bin, Khan, I. A., Yeap, C. S. & Fun, H.-K. (2009*b*). *Acta Cryst.* E**65**, m1624–m1625. 10.1107/S1600536809046935 PMC2971926 21578645

[bb10] Shawkataly, O. bin, Ramalingam, K., Fun, H.-K., Abdul Rahman, A., & Razak, I. A. (2004). *J. Cluster Sci.* **15**, 387–394.

[bb11] Shawkataly, O. bin., Ramalingam, K., Lee, S. T., Parameswary, M., Fun, H.-K. & Sivakumar, K. (1998). *Polyhedron*, **17**, 1211–1216.

[bb12] Sheldrick, G. M. (2008). *Acta Cryst.* A**64**, 112–122. 10.1107/S0108767307043930 18156677

[bb13] Spek, A. L. (2009). *Acta Cryst.* D**65**, 148–155. 10.1107/S090744490804362X PMC2631630 19171970

